# Amino acid metabolism modulates macrophage polarization: implications for autoimmune-related diseases

**DOI:** 10.3389/fimmu.2026.1736082

**Published:** 2026-03-26

**Authors:** Yuzhe Yin, Chaosheng Yan, Xinyu Pei, Jingjing Rao, Ling Wu, Yuzheng Xue, Haowen Sun, Yingyue Sheng

**Affiliations:** 1 Affiliated Hospital of Jiangnan University, Wuxi, Jiangsu, China; 2 Wuxi School of Medicine, Jiangnan University, Wuxi, Jiangsu, China

**Keywords:** arginine metabolism, glutamine metabolism, immunometabolism, metabolic reprogramming, therapeutic targeting, tryptophan catabolism

## Abstract

Amino acid metabolic reprogramming is an important component of immunometabolism. In addition to providing biosynthetic substrates and energetic support for macrophages, distinct amino acid metabolic pathways can also reshape the inflammatory and reparative functional states of macrophages by regulating redox homeostasis, epigenetic modifications, signal transduction, and the accumulation of metabolic intermediates. Despite rapid progress in this field, there remains a lack of systematic integration regarding how key metabolic axes, including arginine metabolism, tryptophan catabolism, and glutamine metabolism, coordinately or antagonistically drive macrophage functional reprogramming, as well as the conservation, heterogeneity, and translational significance of these changes across different autoimmune-related diseases. This review summarizes the roles of arginine, tryptophan, glutamine, branched-chain amino acid, serine/glycine/threonine, aspartate/asparagine, and sulfur-containing amino acid metabolism in the dynamic spectrum of macrophage polarization, and further outlines recent advances in systemic lupus erythematosus, rheumatoid arthritis, inflammatory bowel disease, multiple sclerosis, type 1 diabetes mellitus, psoriasis, autoimmune hepatitis, and vasculitis. This review emphasizes that amino acid metabolism is not an isolated regulatory module, but rather part of an interconnected network that, together with glycolysis, the pentose phosphate pathway, tricarboxylic acid cycle anaplerosis, one-carbon metabolism, and lipid metabolism, determines macrophage fate. Given the existing differences in evidence strength and metabolic phenotypes among *in vitro* systems, animal models, and human studies, caution is still required when extrapolating these conclusions to clinical settings. Overall, therapeutic interventions targeting amino acid metabolism may provide novel biomarkers and treatment strategies for autoimmune-related diseases, but their clinical translation still depends on higher-resolution human validation and mechanism-oriented precision studies.

## 1 Introduction

Autoimmune diseases (AIDs) are a group of chronic inflammatory disorders caused by the breakdown of immune tolerance, including rheumatoid arthritis (RA), systemic lupus erythematosus (SLE), inflammatory bowel disease (IBD), multiple sclerosis (MS), type 1 diabetes mellitus (T1DM), and psoriasis. These diseases are typically characterized by persistent inflammatory responses and tissue damage, and their onset and progression involve the combined effects of genetic susceptibility, environmental factors, and dysregulated immune regulation (1–3). In this process, the innate immune system plays an important role in the formation and maintenance of the inflammatory microenvironment, among which macrophages have become a major focus of research because of their key functions in inflammatory amplification, antigen presentation, and tissue repair (4–6). A growing body of evidence indicates that an imbalance in macrophage functional states is closely associated with the onset and progression of multiple autoimmune diseases.

Macrophages exhibit marked functional plasticity, and their phenotypes can dynamically change in response to microenvironmental signals. Traditionally, macrophages have been classified into classically activated M1 and alternatively activated M2 phenotypes. M1 macrophages are characterized by high inducible nitric oxide synthase (iNOS) expression and the secretion of pro-inflammatory factors, whereas M2 macrophages express molecules such as arginase-1 (Arg1) and interleukin-10 (IL-10) and participate in tissue repair (7–10). However, increasing evidence indicates that macrophages *in vivo* do not exist as fixed binary states, but rather display a continuum of functions shaped jointly by microenvironmental signals, metabolic reprogramming, and epigenetic regulation (10–12). Metabolic reprogramming not only fulfills the demands for energy and biosynthesis, but also directly influences inflammatory gene expression by regulating signaling pathways and chromatin states (13, 14).

**Figure 1:**
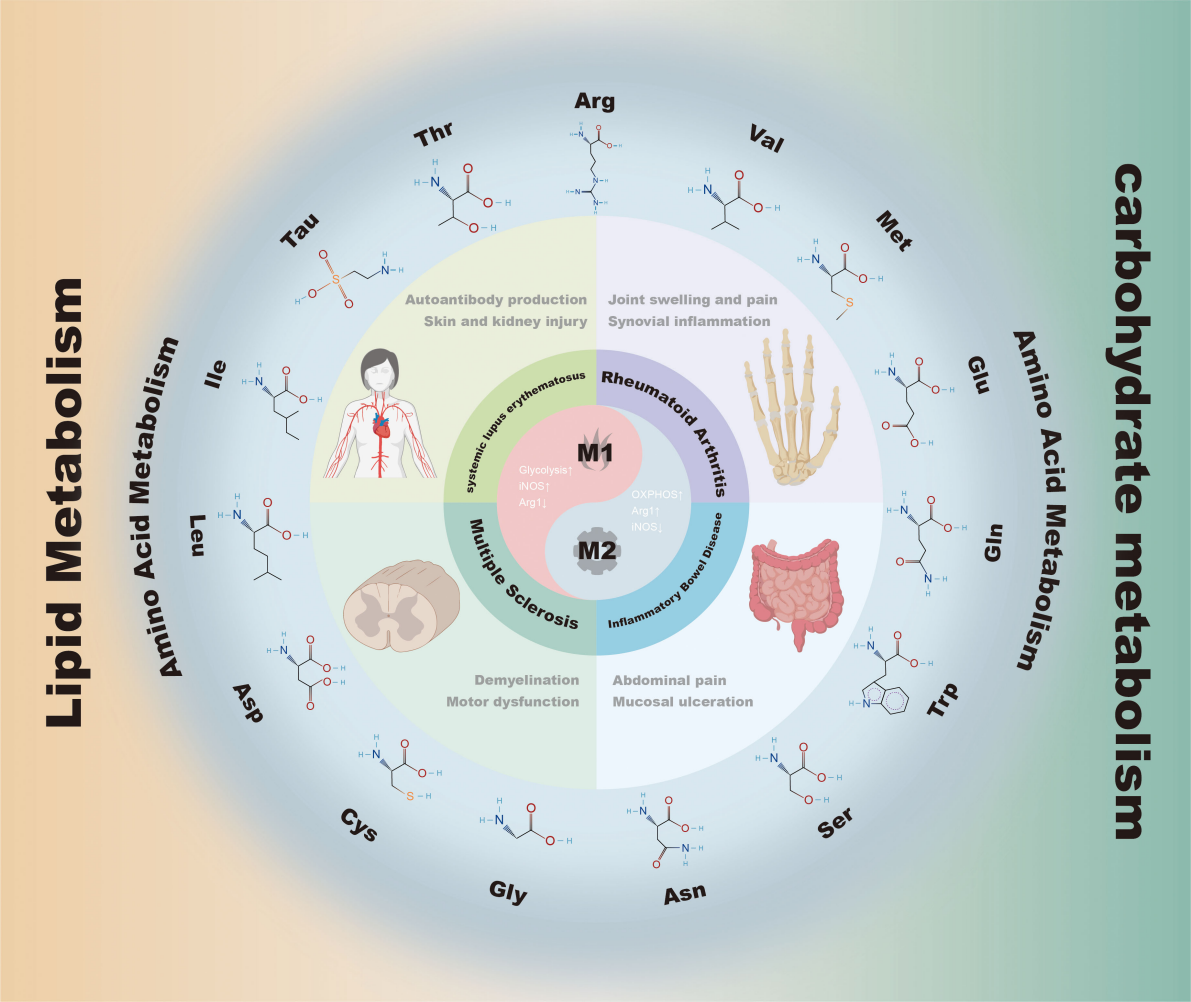
Roles of amino acid metabolism in autoimmune disease-associated macrophage polarization. This image summarizes the key roles of amino acid metabolism in autoimmune disease-associated macrophage polarization. Multiple amino acids, including Arg, Trp, Gln, Ser, Met, Cys, Gly, Leu, Ile, and Val, influence macrophage functional states through metabolic reprogramming, thereby participating in the initiation and regulation of inflammatory responses. Imbalances in different metabolic pathways are closely associated with the immunopathological processes of diseases such as systemic lupus erythematosus, rheumatoid arthritis, inflammatory bowel disease, and multiple sclerosis. The image presents the molecular structures of representative amino acids and their associated diseases, highlighting the interplay between amino acid metabolism and glucose and lipid metabolism in the maintenance of immune homeostasis.

In recent years, studies of immunometabolism have gradually revealed the important role of metabolic reprogramming in regulating immune cell function. Previous studies have mainly focused on the functional divergence between glycolysis and oxidative phosphorylation (OXPHOS) (9). In addition, tricarboxylic acid (TCA) cycle intermediates such as succinate and α-ketoglutarate (α-KG) can influence inflammatory transcriptional programs by regulating hypoxia-inducible factor-1α (HIF-1α) stability or histone demethylase activity (15). These findings indicate that metabolic pathways not only provide energy and biosynthetic substrates for immune cells, but also directly participate in the regulation of inflammatory responses as signaling molecules. In this context, amino acid metabolism has gradually been recognized as a key hub linking energy metabolism and signaling regulation (2, 16, 17). As shown in **Figure 1**, amino acid metabolism constitutes an important regulatory layer connecting immunometabolic reprogramming and macrophage polarization. Arginine regulates the balance between pro-inflammatory and reparative programs through substrate competition between iNOS and Arg1; tryptophan is metabolized by indoleamine 2,3-dioxygenase 1 (IDO1) to generate kynurenine (Kyn) and activate aryl hydrocarbon receptor (AhR) signaling; glutamine metabolism produces α-KG, which participates in TCA cycle anaplerosis and affects epigenetic modifications; and branched-chain amino acids promote inflammatory gene expression through mechanistic target of rapamycin complex 1 (mTORC1) signaling (2, 16, 18). Although related studies continue to accumulate, the current evidence remains fragmented: systematic integration among different amino acid pathways is lacking, the consistency of metabolic features across different experimental models (*in vitro*, animal, and clinical) remains unclear, and metabolic dependence also varies across tissue microenvironments. Therefore, it is necessary to systematically examine how amino acid metabolism regulates macrophage polarization and its role in autoimmune diseases within a network-based framework.

On this basis, this review summarizes the major regulatory mechanisms by which amino acid metabolism shapes macrophage polarization from the perspective of immunometabolic network integration, and further outlines the research progress in different autoimmune diseases. At the same time, this review discusses the potential therapeutic strategies targeting amino acid metabolism and their translational challenges. By integrating the available evidence, it is expected to provide a systematic framework for understanding the metabolic plasticity of macrophages in autoimmune diseases and a theoretical basis for the future development of metabolism-targeted intervention strategies.

## 2 Literature search strategy

To enhance transparency, the literature discussed in this review was identified through searches of PubMed, Web of Science, and Scopus up to January 2026. Search terms combined controlled vocabulary and free-text keywords related to amino acid metabolism, macrophage polarization, immunometabolism, and autoimmune-related diseases, together with specific amino acid pathways and representative disease names. Original studies were prioritized when they directly examined how amino acid metabolic pathways regulate macrophage polarization, inflammatory function, or disease-associated immune phenotypes in *in vitro* systems, animal models, or human samples. Recent reviews were used selectively for conceptual background, theoretical framing, and cross-referencing of relevant primary literature. Studies without clear relevance to macrophage biology, amino acid metabolic regulation, or autoimmune/inflammatory disease contexts were not emphasized in the final synthesis.

## 3 Macrophage polarization and immunometabolism

**Figure 2:**
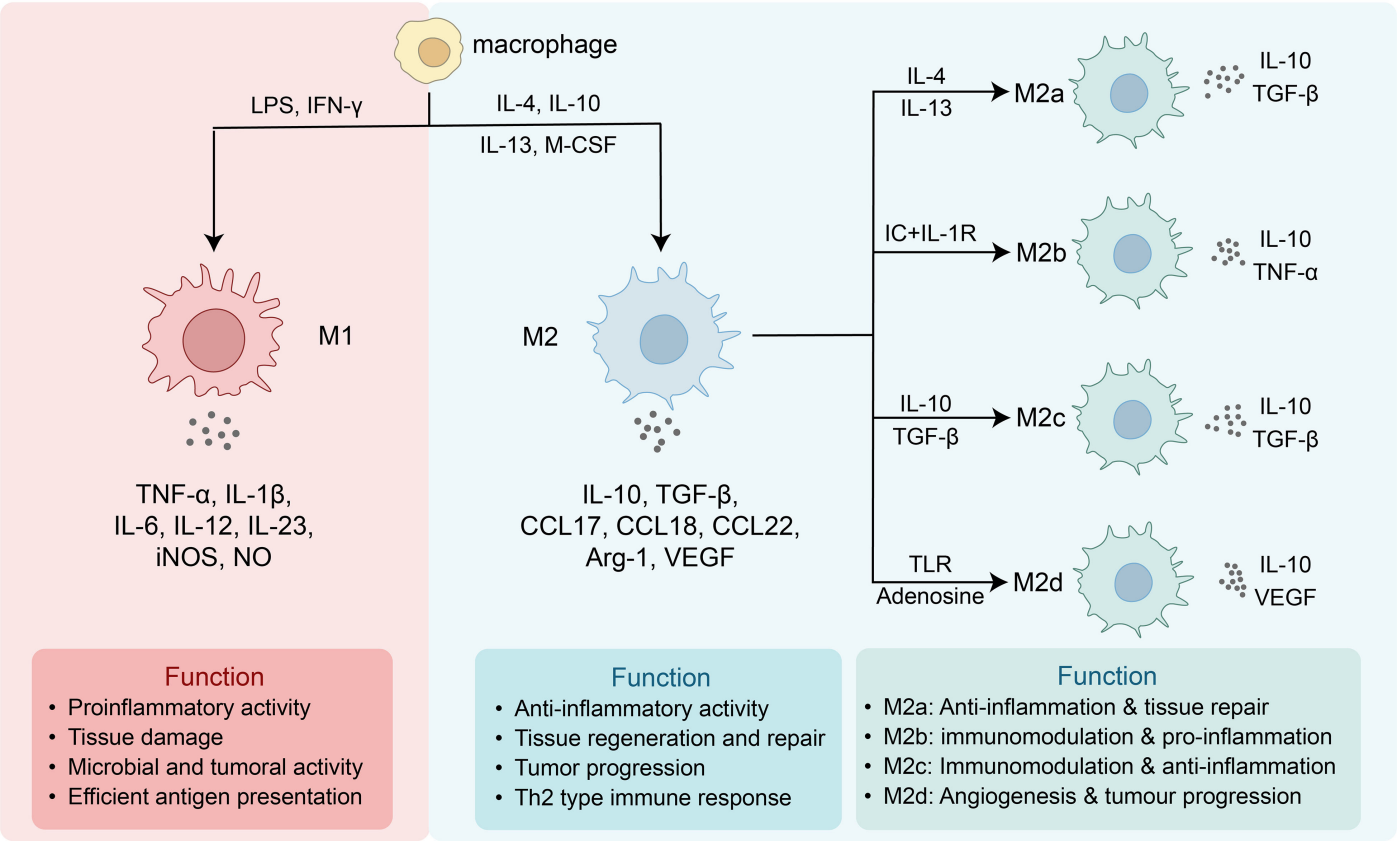
Macrophage polarization and functional heterogeneity. Classically activated M1 macrophages are induced by LPS and IFN-γ and secrete TNF-α, IL-1β, IL-6, IL-12, IL-23, iNOS, and nitric oxide (NO). They exhibit pro-inflammatory, tissue-damaging, antimicrobial, and antitumor activities and are also highly efficient in antigen presentation. Alternatively activated M2 macrophages are generated in response to IL-4, IL-10, IL-13, or macrophage colony-stimulating factor (M-CSF) and secrete IL-10, transforming growth factor-β (TGF-β), C-C motif chemokine ligand 17 (CCL17), CCL18, CCL22, Arg1, and vascular endothelial growth factor (VEGF), thereby exerting anti-inflammatory effects, promoting tissue repair, and facilitating tumor progression. M2 macrophages can be further divided into M2a (induced by IL-4/IL-13, involved in tissue repair and anti-inflammation), M2b (induced by immune complexes and interleukin-1 receptor [IL-1R] stimulation, involved in immune regulation accompanied by inflammatory responses), M2c (induced by IL-10/TGF-β, involved in immune regulation and anti-inflammation), and M2d (induced by toll-like receptor [TLR] signaling or adenosine, involved in angiogenesis and tumor progression).

The functional states of macrophages are not only regulated by microenvironmental inflammatory signals, but are also closely associated with dynamic reprogramming of intracellular metabolic networks, thereby exhibiting a high degree of plasticity. Recent studies have shown that glucose, lipid, and amino acid metabolism form a highly interconnected metabolic network within cells and jointly shape macrophage functional states. Classically activated (M1) macrophages are usually induced by lipopolysaccharide (LPS) or interferon-γ (IFN-γ) and are characterized by high iNOS expression and increased secretion of pro-inflammatory factors such as tumor necrosis factor-α (TNF-α), interleukin-1β (IL-1β), and interleukin-6 (IL-6); alternatively activated (M2) macrophages, by contrast, express molecules such as Arg1, CD206, and IL-10 in response to interleukin-4 (IL-4), interleukin-13 (IL-13), or IL-10 stimulation, and participate in inflammation resolution and tissue repair (7–9). However, increasing evidence indicates that macrophages *in vivo* do not exist as fixed binary states, but rather more closely resemble a continuum of functions jointly shaped by tissue niches, inflammatory stages, and substrate availability, with their phenotypes being collectively determined by microenvironmental signals, metabolic reprogramming, and epigenetic regulation (11, 19–23). Single-cell transcriptomic and metabolic flux analyses further suggest that there is a progressive transition of metabolic trajectories among different polarization states, rather than discrete classifications (12, 24). As shown in **Figure 2**, classically activated M1 macrophages and alternatively activated M2 macrophages differ markedly in inducing signals, secreted factors, and functional characteristics, while M2 macrophages can be further subdivided into multiple subtypes (M2a, M2b, M2c, and M2d). This phenotypic diversity reflects the high adaptability of macrophages to distinct microenvironmental signals and also provides an important basis for metabolic reprogramming-mediated regulation of their functions.

During pro-inflammatory polarization, macrophages typically exhibit a marked increase in glycolytic flux, accompanied by partial “breaks” in the TCA cycle. Tannahill et al. found that LPS stimulation led to an approximately 30-fold accumulation of succinate in mouse bone marrow-derived macrophages (BMDMs), which in turn specifically promoted the transcription and expression of IL-1β by inhibiting prolyl hydroxylase (PHD) and stabilizing HIF-1α (15). At the same time, pentose phosphate pathway (PPP) activity is enhanced, providing NADPH support for reactive oxygen species (ROS) generation (13). In contrast, anti-inflammatory or reparative macrophages rely more on mitochondrial OXPHOS and fatty acid oxidation to meet their energetic demands, while maintaining a relatively intact TCA cycle structure (25). These metabolic changes not only affect energy supply, but also further influence amino acid metabolism and epigenetic regulation by altering the levels of TCA cycle intermediates.

During macrophage metabolic reprogramming, amino acid metabolism does not operate independently, but is embedded within the overall metabolic network. Glutamine is metabolized by glutaminase (GLS) to generate α-KG, which enters the TCA cycle, participates in anaplerotic reactions, and regulates polarization direction. In *in vitro* macrophage polarization models, exogenous α-KG significantly increases Arg1 expression and suppresses iNOS expression, and changes in the α-KG/succinate ratio are closely associated with the expression of M2-related genes (26). Branched-chain amino acids (BCAAs) are metabolized through branched-chain amino acid transaminase (BCAT) and branched-chain α-keto acid dehydrogenase (BCKDH) to generate succinyl-CoA or acetyl-CoA, thereby replenishing the TCA cycle, and their catabolism is associated with a pro-inflammatory phenotype in some models; however, in *in vitro* cultured macrophage models, high concentrations of leucine can enhance anti-inflammatory gene expression through mechanistic target of mTORC1 signaling, which is inconsistent with other theoretical views and suggests that its effects are condition-dependent (27, 28). Aspartate/asparagine participates in the malate-aspartate shuttle and the regulation of nitrogen metabolism (29). These findings suggest that the coupling among glucose, lipid, and amino acid metabolism jointly determines the direction of metabolic flux and the extent to which it supports polarization.

In addition to energy supply and metabolic flux remodeling, amino acid-derived metabolites can also directly participate in signaling regulation. In multiple *in vitro* and animal models, arginine is metabolized by iNOS to generate nitric oxide (NO), thereby enhancing pro-inflammatory responses, whereas its metabolism by Arg1 produces ornithine and polyamines that support tissue repair (10, 30–33). Tryptophan is metabolized by IDO1 to generate Kyn, which can regulate IL-10 expression and influence immune tolerance through AhR signaling (34, 35). Glutamine-derived α-KG not only serves as an intermediate in energy metabolism, but also acts as a cofactor for the Jumonji family of demethylases to participate in transcriptional regulation (26). In addition, S-adenosylmethionine (SAM), generated through the methionine cycle, provides methyl donors for DNA and histone methylation, thereby affecting the expression status of inflammation-related genes (36, 37). Therefore, in macrophages, metabolites function not only as substrates, but also as signaling molecules.

The coupling between metabolism and epigenetic regulation further reinforces the impact of metabolism on the stability of macrophage polarization. In macrophage models, α-KG promotes JMJD3-mediated H3K27me3 demethylation, thereby favoring the expression of M2-related genes, whereas succinate accumulation can inhibit demethylase activity and sustain a pro-inflammatory transcriptional state (26, 38). One-carbon metabolism involving serine, glycine, and methionine provides substrates for the methylation cycle and affects the chromatin accessibility of inflammatory genes (39). These findings suggest that metabolic states not only determine short-term inflammatory responses, but may also shape the persistence of polarization to some extent through epigenetic mechanisms.

It should be noted that most current evidence regarding amino acid-mediated regulation of macrophage polarization is derived from *in vitro* cell models or animal experiments, whereas systematic validation in human clinical samples remains relatively limited. Although the substrate competition between Arg1 and iNOS, as well as the anti-inflammatory effects of α-KG, show a certain degree of consistency across multiple models, branched-chain amino acid (BCAA) and tryptophan metabolism may exhibit divergent or even opposite trends in different disease contexts and tissue microenvironments (40–44). Therefore, when evaluating the translational potential of amino acid metabolism, differences among experimental systems and tissue-specific factors should be fully taken into consideration.

Overall, macrophage polarization is more appropriately understood as the result of metabolic network remodeling, in which glucose metabolism, lipid metabolism, and amino acid metabolism do not operate in isolation, but instead form a highly coupled regulatory system at the levels of metabolic flux direction, signal transduction, and epigenetic regulation. Within this network, different amino acids participate in shaping polarization programs by influencing key nodes such as TCA cycle anaplerosis, mTOR signaling activity, redox balance, and methyl donor cycling. Notably, the roles of different classes of amino acids within this network are not identical. Therefore, to understand the effects of amino acid metabolism on macrophage function, it is necessary to analyze the node-specific roles of different amino acids within a unified metabolic network framework.

## 4 Roles of amino acid metabolism in macrophage polarization

**Figure 3:**
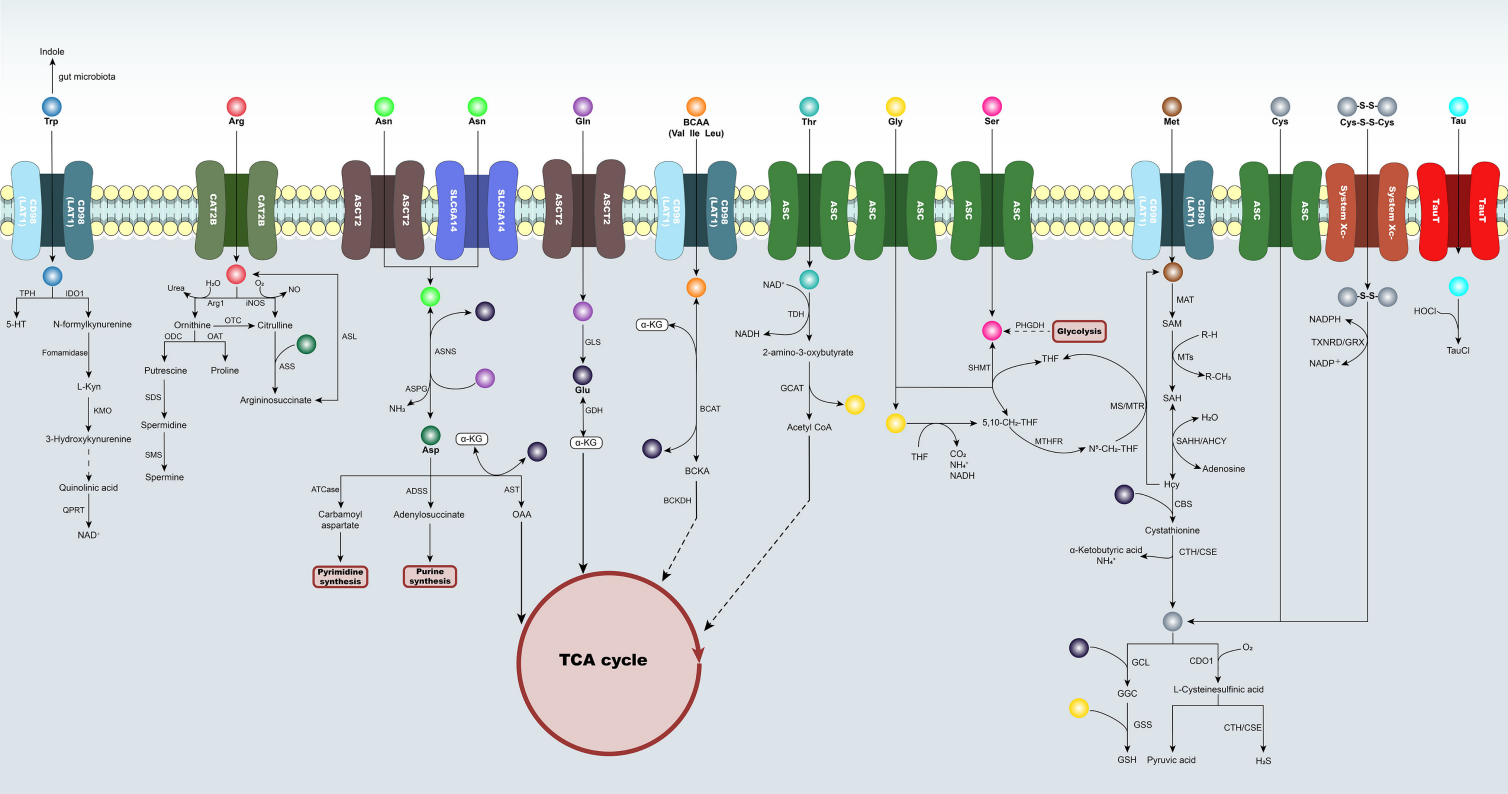
Schematic illustration of the amino acid metabolic network in macrophages. Multiple amino acids enter cells through specific transporters and participate in distinct metabolic pathways. Trp is metabolized through the Kyn pathway (IDO1, KMO, and QPRT) to generate downstream metabolites and regulate immunity; Arg can generate NO through iNOS, or enter polyamine synthesis through arginase/ornithine metabolism; Asn and Asp participate in nucleotide metabolism through purine and pyrimidine synthesis pathways; Gln is converted by GLS into α-KG, which enters the TCA cycle; branched-chain amino acids (BCAAs) are metabolized through BCAT/ BCKDH and are closely associated with energy supply; Ser/Gly/Met participate in one-carbon metabolism and the methylation cycle; and Cys and taurine (Tau) metabolism are involved in glutathione (GSH) synthesis and redox homeostasis regulation. This network integrates the relationships of amino acid metabolism with energy cycling (TCA cycle), nucleotide synthesis, and antioxidant pathways, highlighting its critical roles in immune regulation, inflammatory responses, and tissue homeostasis.

Amino acid metabolism exerts multilevel effects in the regulation of immune cell function. It not only provides carbon and nitrogen sources as well as energetic substrates for cells, but also participates in signal transduction and epigenetic regulation through metabolic intermediates and key enzymatic systems. Given that different amino acids occupy distinct functional levels within the immunometabolic network, this section summarizes the relevant pathways from the perspective of metabolic regulatory mechanisms. Among them, arginine and tryptophan metabolism are mainly manifested as immune regulation mediated by substrate competition or signaling metabolites; glutamine and branched-chain amino acids are more involved in TCA cycle anaplerosis and energy metabolism; whereas pathways involving serine, glycine, and methionine influence epigenetic regulation through one-carbon metabolism and methyl donor cycling. It should be noted, however, that, as shown in **Figure 3**, the amino acid metabolic network is a complex and multidimensional system, and the roles of each amino acid in metabolic reprogramming are also distinct. Different amino acids are interconnected through key nodes such as TCA cycle anaplerosis, nucleotide synthesis, one-carbon metabolism, and redox regulation, and together influence inflammatory transcriptional programs and cellular functional states. Within this network framework, different amino acid pathways may participate in macrophage polarization through distinct metabolic nodes.

### 4.1 Arginine

Arginine (Arg) is one of the amino acids for which the most substantial evidence has accumulated in studies of macrophage polarization. Its key feature lies in the competitive utilization of the same substrate by iNOS (also known as Nos2) and Arg1: the former generates NO and is closely associated with pro-inflammatory effects, whereas the latter produces ornithine and polyamines and participates in tissue repair and immune regulation (31). Therefore, arginine metabolism is often used to understand the metabolic coupling and counterbalance between pro-inflammatory and reparative programs. The dynamic balance between Arg1 and iNOS in arginine metabolism is directly associated with the pro-inflammatory and anti-inflammatory functions of macrophages (32).

Under classical pro-inflammatory stimulation (such as LPS/IFN-γ), iNOS expression is upregulated and catalyzes the conversion of Arg into NO and citrulline. As an effector molecule, NO participates in antimicrobial responses and cooperates with oxidative stress processes to amplify inflammatory signaling, thereby being associated with an M1-like pro-inflammatory program (7, 35, 45). Citrulline directly binds to Janus kinase 2 (JAK2), thereby weakening the binding of JAK2 to IFN-γ receptor 2 (IFNγR2) and signal transducer and activator of transcription 1 (STAT1), inhibiting the activity of the JAK2-STAT1 signaling pathway, and consequently suppressing the pro-inflammatory polarization of macrophages. Argininosuccinate synthetase (ASS1) in the urea cycle controls the activation of inflammatory macrophages by consuming intracellular citrulline (46). In contrast, under reparative signaling conditions such as IL-4/IL-13, Arg1 expression is enhanced, promoting the metabolism of Arg toward ornithine. Ornithine is further converted by ornithine decarboxylase (ODC) into polyamines, such as spermidine and spermine. These metabolites are associated with tissue repair, immune regulation, and the resolution of inflammation, and overall are more consistent with M2-like functional characteristics (31, 47, 48). In addition, spermine has been reported to promote the formation of an APOE^+^ macrophage-like state with immunoregulatory features in mouse macrophage models (30), suggesting that the Arg1-polyamine axis may participate in shaping specific functional subsets. The coupling of Arg metabolism with branches such as proline generation is also considered to be associated with reparative processes (49, 50).

In addition to generating terminal effector molecules, Arg metabolism can also influence inflammatory transcriptional programs through metabolic-signaling coupling. The interactions between citrulline and molecules associated with the JAK/STAT axis, together with the regulatory effects of urea cycle-related enzymes on the activation of inflammatory macrophages, suggest that the iNOS/NO axis may exert feedback regulation on pro-inflammatory signaling in different contexts (51). Therefore, Arg metabolism is more appropriately understood as a regulatory module that can be dynamically reshaped by microenvironmental signals: when the iNOS pathway predominates, it is more likely to drive pro-inflammatory effector output, whereas when the Arg1 pathway predominates, it is more likely to favor tissue repair and immune regulation. The relative weight of these two pathways is influenced by the type of stimulus, substrate availability, and the tissue microenvironment (2, 52).

At the intervention level, regulating the metabolic partitioning of Arg can alter macrophage phenotypes and inflammatory intensity. β2-adrenergic agonists such as terbutaline, or related pharmacological interventions, can modulate Arg metabolism-related processes and shift the polarization balance by affecting pathways such as high-mobility group box 1 (HMGB1) and nuclear factor-κB (NF-κB); nutritional factors may likewise influence this metabolic module and thereby affect polarization bias by altering substrate supply and utilization patterns (48–50). Consistent with this, targeting nodes related to arginine metabolism, including the iNOS/arginase axis, may have potential therapeutic significance in inflammation- and autoimmune-related pathologies (53). Overall, the dynamic allocation of Arg metabolism between the two branches of “NO production-polyamine synthesis” provides a clear metabolic framework for understanding the pro-inflammatory-reparative functional spectrum of macrophages, although its biological effects are markedly context-dependent and should be interpreted in conjunction with the specific experimental system and disease microenvironment. Nevertheless, although the classical Arg1/iNOS competition model provides an important framework for understanding the involvement of arginine metabolism in macrophage polarization, its applicability in the complex pathological environment *in vivo* still requires cautious consideration. ARG1 induction is not absolutely restricted to traditional reparative conditions, and some inflammation-related signals and metabolic environments can also drive its expression. Therefore, arginine metabolism should be understood as a dynamic process jointly regulated by species background, tissue microenvironment, and inflammatory stage, rather than as a static binary switch (11, 53).

### 4.2 Tryptophan

An important feature of tryptophan (Trp) metabolism in macrophage polarization is that its metabolites can directly act as signaling molecules in immune regulation. In addition to serving as a substrate for protein synthesis, Trp is metabolized through the Kyn pathway via IDO1/tryptophan 2,3-dioxygenase (TDO) to generate metabolites such as Kyn, which can influence inflammatory transcription and immune tolerance through signaling axes such as the AhR (2). Compared with pathways that mainly contribute to energy production or anabolic metabolism, Trp metabolism is more prominently characterized by immune regulation at the level of the “metabolite-receptor-transcriptional program” axis, and therefore has important significance in shaping macrophage phenotypes (31).

In the immune microenvironment, Trp can enter the Kyn pathway through IDO1 or TDO, generating Kyn and its downstream metabolites. This pathway can not only trigger amino acid starvation stress through Trp depletion, but also reshape inflammatory transcriptional programs through Kyn-mediated activation of the AhR as a ligand (2, 35, 54). By contrast, the serotonin metabolic pathway of Trp has been less extensively studied in macrophages. Therefore, Trp metabolism is more appropriately understood as a key node linking immunometabolic states with the balance between immune tolerance and inflammation.

At the mechanistic level, the Kyn-AhR axis is regarded as an important pathway through which Trp metabolism influences macrophage phenotypes. As a ligand-dependent transcription factor, AhR activation can induce the expression of multiple immune regulation-related genes and may produce distinct regulatory effects under different ligand profiles and microenvironmental conditions; however, in multiple macrophage research models, it is associated with IL-10 upregulation, suppression of pro-inflammatory signaling, and tolerance-related phenotypes (55, 56). At the same time, Trp depletion itself can activate amino acid stress pathways, such as general control nonderepressible 2 (GCN2)-related responses, and suppress pro-inflammatory reactions, thereby counterbalancing M1-like programs (54). Although indoleamine 2,3-dioxygenase 2 (IDO2) is homologous to IDO1, it differs in catalytic efficiency and tissue distribution, and is considered to potentially participate in Trp metabolic regulation in the context of certain autoimmune diseases (2). It should be emphasized that the immunological effects of Trp metabolism are markedly context-dependent: AhR activation may produce differential outputs under different ligand spectra, cell types, and inflammatory stages. Therefore, the Trp-Kyn-AhR axis may not show completely consistent directions across different disease models, which is also a key point that needs to be interpreted in conjunction with specific systems in the subsequent disease-related sections.

In addition to host enzymatic systems, the gut microbiota can also convert Trp into a variety of indole derivatives, such as indole-3-acetic acid, and these metabolites can likewise act as AhR ligands to participate in the regulation of mucosal immune homeostasis and may alter inflammatory outcomes by affecting the immunoregulatory programs of local macrophages/mononuclear phagocytes (51). Microbiota-derived Kyn and its derivatives are able to recruit GPR35-positive macrophages and participate in mucosal immune homeostasis (57). Therefore, when discussing the effects of Trp metabolism on macrophage polarization, it is necessary to consider the combined shaping of the local ligand spectrum by both the host IDO axis and microbial Trp metabolism.

Overall, Trp metabolism influences the functional spectrum of macrophages through two intertwined pathways, namely the “stress response induced by Trp depletion” and the “AhR signaling mediated by Kyn/indole derivatives.” The overall outcome is often manifested as restriction of pro-inflammatory programs and support of immunoregulatory/tolerance-related phenotypes, although this effect is condition-dependent and influenced by ligand source (host or microbial), inflammatory stage, and the tissue microenvironment. This feature endows the Trp metabolic axis with potential interventional value in autoimmune-related inflammation, while also indicating that its translational application requires careful evaluation of ligand spectra, receptor context, and tissue specificity.

### 4.3 Glutamine

Glutamine (Gln) is mainly converted through GLS/glutamate dehydrogenase (GLUD)-related reactions to generate α-KG, which enters the TCA cycle and provides anaplerotic substrates for oxidative metabolism, while also influencing polarization-related epigenetic regulatory processes through metabolic ratios such as α-KG/succinate (26, 58). Therefore, Gln metabolism is generally regarded as a key pathway linking the state of energy metabolism with the regulation of transcriptional programs, and its effects may vary under different tissue microenvironments or stimulatory conditions.

Glutamine (Gln) metabolism in macrophage polarization is mainly manifested as a composite regulatory axis of “TCA cycle anaplerosis-mitochondrial metabolism-epigenetic coupling.” Its role is usually not reflected by the output of a single effector molecule, but rather by the coordinated shaping of polarization-related transcriptional programs through metabolic flux and key intermediates, particularly α-KG (59). Intracellularly, Gln can be converted by GLS1/GLS2 into glutamate, which is subsequently metabolized by GLUD or through transamination reactions to generate α-KG that enters the TCA cycle, thereby providing anaplerotic substrates for oxidative metabolism and supporting mitochondrial OXPHOS activity (58). In multiple IL-4-induced alternative activation models, insufficient Gln supply or restricted glutaminolysis is accompanied by downregulation of M2-related markers such as Arg1, whereas pro-inflammatory markers such as IL-1β are relatively increased, suggesting that Gln metabolism plays an important supporting role in maintaining reparative programs (26, 58).

α-KG is considered a key hub linking Gln metabolism to phenotypic remodeling (26). On the one hand, as an intermediate of the TCA cycle, α-KG can reflect anaplerotic flux and influence mitochondrial metabolic status; on the other hand, α-KG can also serve as a cofactor for dioxygenases such as the Jumonji family of demethylases and participate in the dynamic regulation of epigenetic marks such as H3K27me3, thereby affecting the expression of M2-related genes (38, 59). Consistent with this, exogenous α-KG supplementation can enhance Arg1 expression and suppress iNOS expression, and changes in metabolic ratios such as α-KG/succinate are closely associated with the activation of M2-related transcriptional programs (26). Further mechanistic studies suggest that α-KG-driven epigenetic remodeling can promote the establishment of alternative activation programs. Within the broader framework of immunometabolism, integrative analyses at the level of metabolic modules also suggest that the glutamine/α-KG axis is an important component in regulating macrophage functional states.

It should be noted that the effects of Gln metabolism on polarization direction are markedly context-dependent. Under different types of stimulation, tissue microenvironments, or stress exposure conditions, enhanced glutaminolysis does not necessarily lead to an anti-inflammatory phenotype. For example, in particulate matter exposure-related models, GLS1-mediated enhancement of glutaminolysis, together with alterations in local Gln/α-KG levels, can be accompanied by enhanced M1-like polarization, and inhibition of GLS1 can alleviate this pro-inflammatory polarization trend (60). In addition, pharmacological or delivery-based interventions targeting Gln metabolism may produce different phenotypic outputs in different systems. For example, in certain tissue microenvironments, combined regulation of glucose metabolism and Gln uptake can promote macrophage phenotypic remodeling (61). These findings suggest that Gln metabolism should be understood more appropriately as a “metabolic network node,” whose effects depend on nutrient availability, mitochondrial functional status, and its mode of coupling with other metabolic pathways, such as glycolysis and TCA cycle interruption and anaplerosis.

Overall, in addition to serving as a carbon skeleton source to replenish the TCA cycle, Gln metabolism supports TCA cycle anaplerosis and OXPHOS while providing key intermediates such as α-KG, thereby participating in metabolism-epigenetic coupling and influencing the positioning of macrophages along the pro-inflammatory to reparative functional spectrum (11, 26). The Gln-α-KG metabolic axis can be regarded as an important hub linking mitochondrial metabolism and epigenetic regulation. At the translational level, the tissue specificity and model dependence of glutamine metabolism suggest that its potential as an interventional target should be stratified according to disease stage and microenvironmental characteristics, so as to avoid overinterpretation of its functional outputs in different contexts.

### 4.4 Branched-chain amino acids

Branched-chain amino acids (BCAAs; Leu, Ile, and Val) in macrophages can not only replenish TCA cycle-related intermediates through BCAT/BCKDH-mediated catabolism, but also influence inflammation-related transcriptional programs through transport and nutrient-sensing pathways, such as mechanistic target of mTORC1 (42, 62). Current studies suggest that the effects of BCAAs on polarization phenotypes may be markedly context-dependent, including factors such as dose, cellular metabolic state, and tissue background. Therefore, they should be interpreted from the dual perspectives of energy coupling and signaling regulation.

The regulatory features of BCAAs in macrophage polarization are mainly reflected in their role as an intersection node linking “nutrient sensing-mTOR signaling-energy metabolic coupling.” After entering cells, BCAAs can be converted by BCAT into branched-chain α-keto acids, which are further catabolized by the BCKDH complex to generate products such as acetyl-CoA, succinyl-CoA, or propionyl-CoA, thereby connecting to the supply of TCA cycle intermediates and mitochondrial oxidative metabolism (62). Therefore, BCAA metabolism may not only influence the direction of metabolic flux and mitochondrial metabolic status by replenishing TCA cycle anaplerotic substrates, but may also reshape inflammatory transcriptional programs by regulating key nutrient-sensing pathways.

Current studies suggest that the effects of BCAAs on macrophage phenotypes are markedly context-dependent and may show bidirectional outcomes across different experimental systems. On the one hand, in some *in vitro* or animal models, enhanced BCAA catabolism has been reported to upregulate certain M2-related markers and support alternative activation programs, whereas inhibition of BCAA metabolism may impair the establishment of M2-like phenotypes (62). On the other hand, exogenous BCAA supplementation can also promote pro-inflammatory responses: for example, in specific models, BCAA loading can activate the IFN-γ receptor 1 (IFNGR1)/Janus kinase 1 (JAK1)/ STAT1 axis and enhance the expression of M1-related genes and the secretion of inflammatory factors (42). This phenomenon, in which “catabolism and input load lead to different outputs,” suggests that the effects of BCAA metabolism on polarization cannot be simply classified as unidirectionally pro-inflammatory or anti-inflammatory, but instead need to be interpreted in the context of nutrient availability, substrate flux, and the coupling state with glycolysis/OXPHOS.

At the molecular level, the coupling of BCAAs with mTORC1 signaling provides a key clue for understanding their pro-inflammatory effects. The CD98 transporter system (SLC7A5/SLC3A2) mediates the entry of leucine and other BCAAs into cells, which can drive mTORC1 activation and enhance glycolytic metabolism, thereby promoting the expression of pro-inflammatory genes such as IL-1β, TNF-α, and C-X-C motif chemokine ligand 10 (CXCL10) (18). Because mTOR signaling, together with TCA cycle intermediates, including succinate-related metabolic stress, participates in maintaining inflammatory programs (33), sustained BCAA influx may amplify pro-inflammatory phenotypes through the cascade of “nutrient sensing-metabolic reprogramming-transcriptional activation.” In addition, BCAA metabolism, particularly leucine, can regulate macrophage inflammatory states through the mTORC1-liver X receptor α (LXRα) pathway (28). Moreover, BCAT1 not only participates in BCAA metabolism, but has also been reported to be associated with the metabolic state of pro-inflammatory macrophages, and inhibition of BCAT1 can reduce the tendency toward M1-like polarization (27). At the network level, BCAAs influence TCA-related metabolic supply at the substrate level, while also affecting mTORC1 and its downstream transcriptional programs at the signaling level, thereby forming a dual-channel mode of regulation involving both metabolism and signaling.

Overall, the role of BCAA metabolism in macrophage polarization is more appropriately defined as an “integrative module of nutrient sensing and energy metabolism,” and its biological output is jointly influenced by dose, metabolic state, inflammatory stage, and the tissue microenvironment. This context dependence also suggests that, when applying BCAA-related targets for disease intervention, it is necessary to distinguish between two different levels of regulatory modes: the mTOR-driven pro-inflammatory effects caused by enhanced BCAA uptake/transport, and the support provided by changes in BCAA catabolic flux to mitochondrial metabolism and reparative programs, so as to avoid conflating results obtained from different experimental systems.

### 4.5 Serine, glycine, and threonine

Serine, glycine, and threonine are closely linked to one-carbon metabolism within the metabolic network and determine the metabolic module of “one-carbon metabolism-nucleotide synthesis-methyl donor generation-redox homeostasis.” Unlike arginine or tryptophan, which are more oriented toward regulation through the “effector molecule/receptor signaling axis,” these amino acids more commonly influence inflammation-related gene expression and epigenetic states by affecting processes such as nucleotide synthesis, NADPH supply, and methyl donor generation (39, 63).

Serine (Ser) can be obtained exogenously or synthesized from glycolytic intermediates through the serine synthesis pathway, in which 3-phosphoglycerate dehydrogenase (PHGDH) is the key rate-limiting step (39). After entering one-carbon metabolism, Ser can promote the generation of SAM, thereby affecting the status of DNA/histone methylation and reshaping inflammatory gene expression programs (64). In polarization-related studies, the effects of Ser metabolism on the M1/M2 balance are not unidirectional. In some systems, inhibition of PHGDH activity or restriction of Ser supply can promote IFN-γ-induced pro-inflammatory programs while suppressing IL-4-driven reparative programs, and the underlying mechanisms may be related to changes in chromatin modifications caused by insufficient methyl donor availability as well as reprogramming of the JAK/STAT axis (63). However, other studies have suggested that PHGDH inhibition may limit IL-1β production through NAD^+^ accumulation and its downstream sirtuin 1 (SIRT1)/SIRT3 axis, thereby exhibiting an inhibitory effect on inflammatory output (65). This discrepancy suggests that the functional output of Ser metabolism may be influenced by the type of stimulus, the availability of metabolic substrates, mitochondrial status, and the analytical endpoint used, including inflammatory factors, metabolic flux, or phenotypic markers. Therefore, when interpreting the relationship between Ser metabolism and polarization, the experimental system and evaluation criteria need to be clearly defined.

Glycine (Gly) is tightly coupled to Ser metabolism. It can participate in one-carbon metabolism through serine hydroxymethyltransferase (SHMT) to support nucleotide synthesis, and can also serve as a component of glutathione (GSH) in maintaining cellular redox homeostasis (66). Cell-based studies suggest that Gly-related regulation is more often reflected in its effects on reducing capacity and the stress threshold: by influencing GSH supply and ROS stress, Gly may indirectly alter the extent of pro-inflammatory signaling amplification and phenotypic stability (66). In addition, Gly has been reported to promote an M2-like phenotypic tendency through the regulation of specific microRNAs, such as miR-301a, suggesting that its effects may also involve the level of post-transcriptional regulation (66). Overall, the effects of Gly in polarization are more inclined toward “modular coordination,” that is, supporting or restricting specific phenotypic programs through one-carbon metabolism and antioxidant networks, rather than acting as a single determining factor.

Threonine (Thr) can likewise enter the one-carbon metabolic network and can be converted by threonine dehydrogenase (TDH) into intermediates such as acetyl-CoA, thereby providing a link between energy metabolism and epigenetic regulation (67). In some studies, the Thr metabolism-related product α-aminobutyric acid (AABA) has been shown to suppress the expression of pro-inflammatory macrophage-related genes and to jointly restrain inflammatory responses through metabolic reprogramming and EZH2-dependent epigenetic regulation, suggesting that the effects of amino acid metabolism on macrophage function may also extend to the level of metabolism-epigenetic coupling (67). Because Thr also shares the one-carbon metabolic module with Ser/Gly, its role in macrophages is more likely to be reflected in the indirect regulation of the “methyl donor-chromatin state-inflammatory transcriptional program” axis, although evidence in this area remains relatively limited.

Overall, the effects of Ser/Gly/Thr metabolism on macrophage polarization are mainly mediated by changes in one-carbon metabolic flux, methyl donor availability, and redox homeostasis, and the resulting outcome is often manifested as “threshold regulation” of inflammatory transcriptional programs and alterations in phenotypic stability. The inconsistent directions reported in existing studies suggest that this module is highly context-dependent. Therefore, when citing specific conclusions in subsequent sections, they should be interpreted in conjunction with the stimulation conditions, the mode of metabolic intervention, and the experimental system from which the findings were derived.

### 4.6 Aspartate/asparagine

Aspartate (Asp) and asparagine (Asn) are tightly coupled in nitrogen metabolism and mitochondrial shuttle systems. Asp participates in the malate-aspartate shuttle, linking energy metabolism with nitrogen metabolism, and is also required for rapidly proliferating inflammatory cells under inflammatory conditions because of its involvement in nucleotide synthesis; Asn is generated through catalysis by asparagine synthetase (ASNS) and can influence cellular metabolic states through amino acid transport and nitrogen balance (68, 69).

Aspartate (Asp) and asparagine (Asn) are more often manifested in the macrophage metabolic network as an associated module of “mitochondrial shuttle-nitrogen metabolism-nucleotide synthesis,” rather than as a pathway characterized by the output of a single effector molecule. Asp is not only one of the key intermediates in the TCA cycle and the urea cycle, but can also participate in the transfer of reducing equivalents between the cytoplasm and mitochondria through the malate-aspartate shuttle via specific Asn transporters, thereby influencing mitochondrial metabolic status and inflammatory metabolic reprogramming (68). Under pro-inflammatory stimulation, the association between changes in Asp levels and inflammatory factor production has attracted attention in multiple studies; for example, elevated Asp may be accompanied by enhanced IL-1β production and promote an M1-like polarization tendency, and the underlying mechanism may be related to enhanced HIF-1α stability and activation of inflammasome-related pathways (70). It should be emphasized that this effect does not necessarily mean that Asp itself is a “pro-inflammatory factor,” but more likely reflects its position in the coupling of energy metabolism and metabolic signaling: when shuttle activity and anabolic demand increase, Asp supply may become one of the limiting factors supporting pro-inflammatory transcription and effector molecule synthesis.

Asn is generated from Asp and glutamine through catalysis by ASNS. In addition to serving as a nitrogen source for protein synthesis, it can also alter cellular metabolic states by affecting transmembrane amino acid transport and nitrogen balance (68). In macrophages, Asn-related regulation shows a certain bidirectional nature. On the one hand, both Asn and Asp have been associated with enhanced pro-inflammatory programs in some systems, as reflected by increased production of inflammatory factors or upregulation of M1-like phenotypes (70). On the other hand, transporter-mediated Asn uptake, such as that mediated by SLC6A14, has been reported to regulate inflammatory transcription through mechanisms including histone phosphorylation and to suppress the tendency toward M1-like polarization (68). In addition, the Asp-derived metabolite N-acetylaspartate (NAA) has in some contexts been proposed to act as a signaling-related molecule that supports the maintenance of M2-like functions, suggesting that Asp/Asn metabolism may simultaneously connect different levels of pro-inflammatory effects and reparative programs (71).

Overall, the effects of Asp/Asn metabolism on macrophage polarization are more likely to be mediated through the indirect regulation of metabolic capacity and metabolic signaling. Current evidence suggests that its functional outputs are markedly context-dependent. Therefore, when discussing these pathways in the context of specific disease models, factors such as cell type, tissue microenvironment, substrate availability, and transport status should be considered simultaneously, so as to avoid extrapolating directional results obtained in a single system to the whole.

### 4.7 Sulfur-containing amino acids

Metabolic pathways related to sulfur-containing amino acids, represented mainly by methionine (Met), cysteine (Cys), and their derivatives such as taurine (Tau), can respectively connect methyl donor cycling and redox homeostasis regulation: Met affects methylation capacity through SAM generation, Cys participates in redox balance and signaling regulation through GSH/H_2_S, and Tau is associated with antioxidation and mitochondrial homeostasis (36, 72–77). Given that these pathways converge on the intersecting regulatory level of “methylation potential-oxidative stress-inflammatory signaling,” this section discusses their representative mechanisms and their effects on macrophage phenotypes together.

Sulfur-containing amino acid-related metabolic pathways, mainly including methionine, cysteine, and taurine, are characterized in macrophage polarization by the intersecting properties of “methyl donor cycling-transsulfuration-redox homeostasis.” Methionine (Met) not only serves as a substrate for protein synthesis within cells, but also generates SAM through the methionine cycle. SAM is the major methyl donor for multiple methylation reactions and thereby influences DNA/histone methylation status and inflammatory transcriptional programs (36, 37). In multi-omics studies and functional experiments, active Met metabolism and its associated gene networks have been linked to macrophage states with immunosuppressive/reparative tendencies, suggesting that the “Met-SAM axis” may participate in shaping phenotypic stability by regulating methylation potential (37, 78). It should be noted that the effects of Met metabolism on polarization are more often reflected in the regulation of the “plasticity and maintenance” of transcriptional programs, rather than in the immediate output of a single inflammatory mediator. Therefore, its directionality may vary depending on the stimulatory context and tissue microenvironment.

Cysteine (Cys) is an important fulcrum of redox homeostasis. As one of the rate-limiting substrates for the synthesis of GSH, which maintains redox balance, Cys availability and GSH levels jointly influence ROS stress and the extent of inflammatory signal amplification, thereby indirectly shaping the pro-inflammatory or reparative functional spectrum (11). Within the sulfur-containing amino acid network, enzymes related to the transsulfuration pathway, such as cystathionine β-synthase (CBS) and cystathionine γ-lyase (CSE), can also convert Cys into hydrogen sulfide (H_2_S), which, as a gaseous signaling molecule, can regulate multiple inflammation-related pathways and affect macrophage phenotypes (74–77). Previous studies have shown that H_2_S donors or related release systems, such as S-propargyl-cysteine (SPRC), can suppress the expression of M1-related inflammatory factors and promote the formation of M2-like phenotypes, suggesting that the “Cys-GSH/H_2_S axis” may jointly restrain pro-inflammatory programs by lowering the oxidative stress threshold and modulating signal transduction (79).

Taurine (Tau) is abundant in macrophages and is mainly acquired through uptake, and its function is more closely related to antioxidation and the maintenance of organelle homeostasis. In *in vitro* or animal models, Tau can suppress LPS/IFN-γ-induced M1-like polarization and promote the upregulation of M2 phenotypic markers through regulatory axes associated with the methylation cycle and mitophagy (72). These findings suggest that, beyond the “substrate-energy” dimension, sulfur-containing amino acid-related metabolites can also regulate macrophage functional states in chronic inflammatory environments by affecting processes such as oxidative stress, protein modification, and mitochondrial homeostasis.

Overall, sulfur-containing amino acid metabolism can be more appropriately viewed as an integrated module of “methylation potential and redox homeostasis”: the Met-SAM axis is mainly associated with the plasticity of epigenetic and transcriptional programs, the Cys-GSH/H_2_S axis primarily influences oxidative stress and the threshold of inflammatory signaling, and Tau is further linked to antioxidation and the regulation of mitochondrial homeostasis. Current evidence suggests that the output of this module is context-dependent and may be particularly influenced by substrate uptake, oxidative stress within the tissue microenvironment, and the inflammatory stage. Therefore, these background factors should be considered simultaneously when investigating this module in specific disease models.

**Figure 4:**
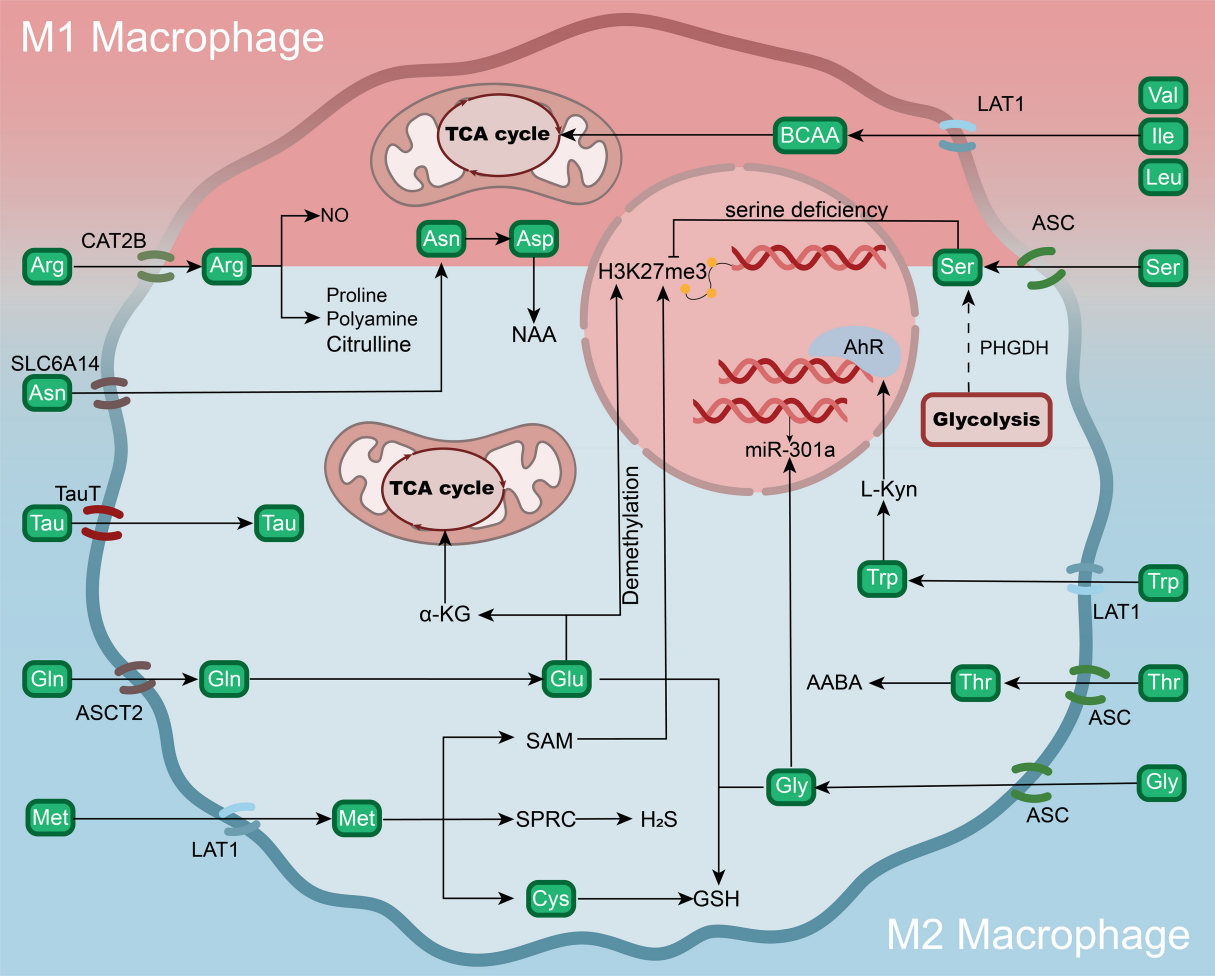
Mechanisms by which amino acids and their metabolites regulate macrophage polarization. Different amino acids enter macrophages through specific transporters and participate in metabolic networks to regulate M1/M2 polarization states. Arginine (Arg) generates NO through iNOS or enters the polyamine and citrulline pathways; glutamine (Gln) is transported through ASCT2, enters the α-KG cycle, and promotes DNA demethylation; serine (Ser) deficiency can affect epigenetic modifications and AhR signaling through the PHGDH/glycolysis pathway; tryptophan (Trp) is metabolized into Kyn, which activates AhR and regulates miR-301a expression; cysteine (Cys) and its downstream metabolites, GSH and hydrogen sulfide (H2S), participate in redox balance and anti-inflammatory responses; methionine (Met) participates in epigenetic regulation through the SAM-mediated methylation cycle; and branched-chain amino acids (BCAAs) enter cells through LAT1 and replenish TCA cycle-related energy supply. The coordinated actions of different amino acid metabolic pathways and their metabolites together constitute the metabolic regulatory network of macrophage polarization.

As shown in **Figure 4**, different amino acid pathways do not act independently during macrophage polarization, but instead form a highly coupled regulatory network through multiple levels, including metabolic flux direction, signal transduction, and epigenetic regulation. Within this framework, the roles of different amino acid pathways in the metabolic network can be understood as differential regulation of key nodes, such as TCA cycle anaplerosis, mTOR signaling, redox balance, or methyl donor cycling. However, it should be noted that current evidence regarding amino acid metabolism-mediated regulation of macrophage polarization is derived mainly from *in vitro* macrophage models and animal experiments, whereas systematic validation in human diseases remains relatively limited. At the same time, research on amino acid metabolism in macrophages has still been focused primarily on amino acids such as Arg and Trp, while studies on other amino acids remain relatively limited, and their specific mechanisms still require further elucidation.

## 5 Research progress on amino acid metabolism in autoimmune-related diseases

The studies described above indicate that different amino acid metabolic pathways can influence macrophage polarization through mechanisms such as substrate competition, signal transduction, TCA cycle anaplerosis, and epigenetic regulation. With the accumulation of related studies, current evidence has been concentrated mainly in diseases that have been more extensively investigated, such as SLE, RA, IBD, and MS. However, in diseases such as T1DM, psoriasis, autoimmune hepatitis, and vasculitis, studies have also suggested that amino acid metabolic reprogramming may participate in the regulation of macrophage function and may exhibit directional effects and context-dependent differences that vary with tissue niche, stimulus spectrum, and disease stage (31, 59, 80–82). Compared with SLE, RA, IBD, and MS, direct mechanistic evidence for amino acid metabolism-mediated macrophage polarization remains relatively limited in other autoimmune diseases. Therefore, at this stage, the available findings are more supplementary and indicative in nature, rather than constituting an established unified mechanistic framework. The sources and strength of evidence also vary across different diseases, including *in vitro* macrophage experiments, animal models, and limited studies in human samples. Accordingly, the translational significance of different conclusions should be interpreted cautiously in light of the specific research context.

SLE is a typical autoimmune disease characterized by widespread immune dysregulation and multiorgan damage. In recent years, multiple *in vitro* and animal studies have revealed a key regulatory role of amino acid metabolism in macrophage polarization in SLE. In cell and animal models, amino acid metabolism not only provides substrates for energy production and biosynthesis, but also regulates the differentiation of macrophages toward M1 or M2 phenotypes by modulating signaling pathways such as STAT1, mechanistic target of rapamycin (mTOR), and NF-κB, as well as by affecting epigenetic regulation (59). In patients with SLE, increased Trp degradation is significantly associated with high IFN-γ production and correlates with disease activity, suggesting that IDO1 may be one of the key factors influencing macrophage polarization-related pathogenicity in SLE (83). Studies have shown that the expression of Arg1 and IDO1 is significantly altered in peripheral monocytes and macrophages from patients with SLE. High Arg1 expression is associated with M2 polarization and exerts immunosuppressive effects, whereas low IDO1 expression is closely associated with loss of tolerance and enhanced pro-inflammatory responses (2). However, some studies have found that Kyn/Trp levels are elevated in SLE, accompanied by increased IDO activity, and are correlated with disease activity, which may result from mTOR activation induced by elevated Kyn levels (40, 41, 43). Activation of the toll-like receptor (TLR)/AMP-activated protein kinase (AMPK)/mTORC1 signaling pathway is critical for the induction of M1 macrophages and interleukin-12 (IL-12) secretion, and high IDO expression has also been observed in M1 macrophages (44). These findings suggest that abnormal tryptophan metabolism may enhance M1 macrophage polarization. Davison et al. found that patients with SLE and renal involvement showed clinical improvement after appropriate inhibition of IDO (84). The specific roles and mechanisms of Trp and other amino acids still require further investigation, and there may be a balancing regulatory mechanism whereby IDO1 can be induced by the pro-inflammatory signals that it suppresses (85). Overall, among the associations between amino acid metabolic abnormalities and macrophage polarization imbalance in SLE, the Trp-Kyn-IDO1/AhR axis remains the most strongly supported by current evidence, although its directionality is not completely consistent across human studies and experimental models. Future studies are needed, particularly in clearly defined organ-involved settings such as lupus nephritis, to combine single-cell analysis, spatial transcriptomics, and metabolic flux analysis in order to distinguish the pathogenic driving role of metabolic alterations from their role as accompanying biomarkers.

In RA, synovial macrophages are considered an important cell population driving chronic inflammation and joint destruction, and their metabolic reprogramming is closely associated with inflammatory phenotypes. The effects of amino acid metabolism on macrophage polarization in RA are gradually becoming a research focus. Single-cell omics and metabolic reprogramming studies have revealed the presence of macrophage subsets with distinct metabolic features in RA. For example, synovial macrophages from patients with ACPA-negative RA exhibit reprogramming of multiple amino acid metabolic pathways, and this metabolic phenotype is closely linked to disease progression (86). Metabolomic studies have shown that amino acid metabolic profiles are markedly disrupted in collagen-induced arthritis (CIA) mice, and abnormalities in pathways involving glutamine, arginine, and serine are closely associated with inflammatory status. During the early inflammatory phase, no joint swelling was observed in CIA mice, and metabolic changes mainly involved arginine biosynthesis, arginine/proline metabolism, phenylalanine/tyrosine/tryptophan biosynthesis, and GSH metabolism. During the peak inflammatory phase, CIA mice developed severe arthritic symptoms and showed broader metabolic alterations in valine/leucine/isoleucine biosynthesis, phenylalanine/tyrosine/tryptophan biosynthesis, and arginine biosynthesis. The reduction of specific amino acids, such as glycine, serine, and proline, in the early stage may lead to an imbalance in macrophage polarization, thereby enhancing inflammatory responses in CIA mice (87). In RA, arginine shifts energy metabolism from glycolysis to OXPHOS, thereby leading to metabolic reprogramming of osteoclasts and a bias toward M2 polarization, ultimately suppressing arthritis, blocking osteoclast formation, and preventing inflammatory bone loss and erosion (88). In follicular macrophages from RA, upregulation of the BCAA transporter SLC7A5 is closely associated with activation of the mTORC1 signaling pathway, which in turn promotes M1 macrophage polarization and arthritic tissue damage (18). In addition, inhibition of BCAA metabolism, for example by suppressing BCAT1, can significantly alleviate arthritic symptoms in RA mice (87). These findings indicate that BCAA metabolism and its regulatory mechanisms may represent potential therapeutic targets in RA, particularly in relation to macrophage polarization. Amino acid metabolism is not only a key determinant of macrophage polarization in RA, but also provides new targets for disease treatment. Future studies need to further integrate metabolomics, single-cell transcriptomics, and clinical trial data to clarify the functions of different amino acid pathways across distinct RA subtypes and disease stages, thereby laying the foundation for precision metabolism-targeted intervention strategies.

In IBD, intestinal mucosal macrophages play a key role in maintaining intestinal immune homeostasis and inflammatory responses, and their functional states are markedly influenced by metabolic reprogramming. Amino acid metabolism plays a critical regulatory role in macrophage polarization. Studies in DSS and TNBS mouse models as well as *in vitro* experiments have shown that amino acids not only serve as substrates for energy production and biosynthesis, but also influence the M1/M2 macrophage balance through metabolic pathways, thereby regulating intestinal inflammation. In patients with IBD, intestinal macrophages exhibit dual metabolic characteristics: during the acute phase, macrophages tend to undergo M1 polarization, accompanied by elevated NO levels; whereas during the chronic phase, M2 markers, such as Arg1, are increased, but may promote intestinal fibrosis (89, 90). The metabolic state of macrophages not only affects the initiation and maintenance of inflammation, but may also play a role in intestinal repair and fibrosis. Arginine metabolism plays a double-edged role in regulating macrophage function: on the one hand, arginine is metabolized by iNOS into NO to promote M1 responses; on the other hand, arginine can generate polyamines through the arginase pathway, thereby promoting the M2 phenotype (91). Interventions such as resveratrol have been shown to alleviate DSS-induced colitis by regulating the microbiota-arginine metabolism axis, increasing M2 macrophage markers, and decreasing M1 macrophage markers (92). The Trp-Kyn-AhR pathway is impaired in IBD. Studies have shown that in mice with DSS-induced acute colitis, IDO1 expression and the Kyn/Trp ratio are significantly increased, whereas they decline during the chronic phase; administration of an IDO1 positive regulator during the chronic stage can promote mucosal healing and attenuate inflammation (93). Reduced IDO1 is associated with the progression of colonic inflammation in mice, and supplementation with tryptophan (Trp) or an AhR agonist can ameliorate intestinal inflammation, suggesting that this pathway may have important clinical value in the treatment of IBD (94, 95). In addition, non-essential amino acids such as glycine, serine, and related metabolites also play roles in macrophage polarization. For example, glycine signaling can alleviate pathological injury by inhibiting pro-inflammatory pathways in TNBS/DSS colitis and regulating the gut microbiota (66). The mechanisms by which amino acid metabolism regulates macrophage polarization in IBD are not confined to cells alone, and the abundant gut microbiota provide a basis for microbiota-cell metabolic interactions. These studies provide experimental support for the development of IBD intervention strategies targeting amino acid metabolism.

Amino acid metabolism plays a central role in macrophage polarization in MS and its animal model, experimental autoimmune encephalomyelitis (EAE). A growing number of studies have shown that different amino acid metabolic pathways determine the balance between pro-inflammatory (M1) and anti-inflammatory (M2) macrophage states by regulating energy supply and immune signaling pathways, thereby influencing disease progression. Arginine metabolic regulators such as spermidine have been shown to suppress M1 macrophage-associated cytokines while inducing M2 macrophage-related markers, significantly alleviating EAE symptoms, whereas Arg1 inhibitors can reverse these effects (96). Mice with AhR deficiency in microglia, a macrophage population in the brain, develop more severe EAE, with increased expression of pro-inflammatory genes and loss of IL-10 and transforming growth factor-α (TGF-α) expression. A tryptophan-deficient diet leads to more severe EAE, whereas tryptophan supplementation reverses this effect, suggesting that tryptophan metabolites exert their functions through activation of AhR in microglia. In the EAE model, microbiota-induced elevation of L-Kyn significantly increases the infiltration of AhR-positive macrophages into the central nervous system, thereby alleviating neuroinflammation and demyelination (31). Another study found that loss of IDO1 expression in the EAE model enhances the pro-inflammatory tendency of M1 macrophages, reduces IL-10 levels, and aggravates disease severity (97). Dietary tryptophan (Trp) supplementation can reverse symptoms in mice with activated AhR, indicating that Trp metabolism is critical for the tolerance of macrophages and microglia (98). In MS and EAE models, a reduced serum L-Kyn/Trp ratio suggests impaired IDO1 function and is closely associated with disease activity (97). These findings provide potential biomarkers for the early diagnosis and treatment of MS and EAE, and further suggest that targeting IDO1 or its metabolites may effectively regulate macrophage polarization and thereby improve disease prognosis. Aromatic amino acids (AAAs) influence the transition of macrophages and microglia toward neurotoxic or protective phenotypes by regulating the AhR pathway (82). Other amino acids, such as serine, cysteine, and phenylalanine, have also gradually attracted attention. Through multiple pathways, amino acid metabolism determines the direction of macrophage polarization in MS, and together these mechanisms highlight the dual role of amino acid metabolism in MS pathogenesis and as a therapeutic target. In the future, targeting amino acid metabolic pathways may become a new strategy for regulating macrophage polarization, promoting neural repair, and limiting inflammatory damage.

In other autoimmune diseases, amino acid metabolic reprogramming likewise participates in the formation of the inflammatory microenvironment by regulating macrophage polarization. For example, in T1DM, infiltrating macrophages within the insulitic microenvironment are considered important effector cells contributing to β-cell injury, and their functional states are closely associated with arginine metabolism. In macrophages, arginine mainly exerts its effects through two competing metabolic pathways. On the one hand, iNOS converts L-arginine into NO and citrulline, thereby driving pro-inflammatory M1-like polarization; on the other hand, Arg1 metabolizes arginine into ornithine and polyamines, thereby promoting an anti-inflammatory or tissue-reparative M2-like phenotype. Studies have shown that, in both non-obese diabetic (NOD) mouse models and patients with T1DM, dysregulated arginine metabolism is closely associated with increased macrophage NO production and is linked to pancreatic β-cell injury and disease progression (99).

A similar metabolism-polarization mechanism has also been demonstrated in psoriasis. A large number of activated macrophages are present in psoriatic lesions, and their polarization states play an important role in maintaining inflammation. In a T cell-mediated chronic psoriasiform inflammation model, investigators observed the coexistence of pro-inflammatory macrophages expressing iNOS and alternatively activated macrophages expressing Arg1 in lesional tissues, suggesting that the partitioning of arginine metabolic pathways participates in regulating macrophage phenotypes and the intensity of inflammatory responses. Further studies demonstrated that sustained macrophage activation is essential for the maintenance of psoriasiform inflammation (100).

In autoimmune hepatitis (AIH)-associated inflammation, the polarization of hepatic macrophages, including Kupffer cells and monocyte-derived macrophages, is likewise regulated by arginine metabolism. In a concanavalin A (ConA)-induced immune hepatitis model, iNOS-related signaling pathways can regulate macrophage polarization through mechanisms involving mitogen-activated protein kinase (MAPK) pathways and thereby influence the intensity of inflammatory responses, indicating that a shift of arginine metabolism toward the NO-generating pathway may promote pro-inflammatory macrophage responses and aggravate hepatic immune injury (101).

ANCA-associated vasculitis (AAV) is a group of autoimmune diseases characterized by small-vessel inflammation, in which monocytes and macrophages play important roles in vascular wall inflammation and tissue injury. Pathological studies have shown that iNOS-expressing macrophages can be detected in AAV lesions, suggesting that the metabolic reprogramming of arginine toward the NO pathway is closely associated with the activation of pro-inflammatory macrophages (102). In addition, interactions between T cells and macrophages can influence macrophage activation and may be associated with changes in iNOS expression and inflammatory responses in the vascular wall, thereby contributing to the immunopathogenesis of vasculitis (103).

In experimental autoimmune uveitis (EAU), intraocular inflammation is likewise closely associated with macrophage polarization. Studies have shown that modulation of macrophage polarization states can significantly influence the severity of inflammation in EAU. For example, progranulin has been shown to suppress inflammation and promote the transition of macrophages toward an anti-inflammatory phenotype in the EAU model, thereby alleviating retinal injury (104). In addition, natural products such as morroniside and baicalin have also been reported to improve inflammatory manifestations in EAU by regulating signaling pathways related to macrophage polarization (105, 106).

In autoimmune hemolytic anemia (AIHA), macrophage-mediated erythrophagocytosis is an important effector process in disease development. Studies have shown that erythrophagocytosis can induce marked metabolic reprogramming in macrophages and affect their inflammatory phenotypes and immune functions (107). In addition, the key roles of macrophages in erythropoiesis and erythrocyte clearance have been systematically reviewed, and these processes are both accompanied by substantial metabolic adaptive changes, providing a theoretical basis for macrophage metabolism-function coupling in AIHA (108).

It should be noted that although a growing number of studies have shown that amino acid metabolic reprogramming is widely present in the inflammatory microenvironment of multiple autoimmune diseases and is closely associated with the functional states of macrophages, current evidence also suggests that, in most diseases, alterations in amino acid metabolism are not necessarily primary drivers of disease onset, but may more likely reflect metabolically adaptive changes during immune cell activation and polarization. For example, the partitioning of arginine metabolism (the iNOS/Arg1 axis) or alterations in the Trp-Kyn pathway observed in various inflamed tissues often occur in parallel with M1/M2-like macrophage polarization states, and therefore may more likely serve as metabolic manifestations of inflammatory status and immune cell functional remodeling. From this perspective, amino acid metabolism not only participates in the regulation of inflammatory responses, but may also serve as an important metabolic biomarker reflecting changes in the immune microenvironment. Future studies are still needed to further clarify the causal relationships of amino acid metabolism in autoimmune diseases and its hierarchical position in immune regulation through more systematic genetic studies, metabolic flux analyses, and single-cell multi-omics approaches, so as to distinguish its specific role as a disease driver or as a passenger/metabolic marker. At the same time, given the close coupling between amino acid metabolism and macrophage polarization, targeting related metabolic pathways may still provide new strategies for modulating inflammatory responses.

Overall, the evidence in this section supports the view that amino acid metabolism does not merely provide energy and biosynthetic substrates, but instead shapes the functional spectrum of myeloid cells through several reusable network-level nodes. Substrate partitioning determines the allocation of inflammatory mediators and reparative substrates through competing pathways such as iNOS/Arg1; the glutamine-TCA anaplerosis axis together with nutrient-sensing pathways jointly sets the metabolic threshold for pro-inflammatory or tolerance/reparative programs; and one-carbon/methyl donor metabolism together with redox homeostasis influences the stability and reversibility of polarization-related transcriptional and epigenetic programs. Cross-disease comparisons suggest that these modules recur across different organ niches, but their net effects are markedly context-dependent and are jointly regulated by disease stage (acute inflammation versus chronic tissue remodeling), tissue compartment (peripheral blood versus lesional tissue), as well as the spectrum of stimuli and therapeutic exposure, thereby leading to inconsistent directional outcomes of the same pathway across different models and time windows. Current studies still commonly face limitations in causal inference and model systems: most findings are derived from *in vitro* induction systems or single animal models, making it difficult to simultaneously resolve the coordinated influence of tissue substrate availability, intercellular metabolic division of labor, and local cytokine networks on metabolic pathways; clinical studies are mostly cross-sectional and correlative, lacking stratification by dose, time window, and tissue source, as well as dynamic follow-up to support testable mechanistic inferences. More explanatory evidence in the future will require the simultaneous reporting of metabolites/metabolic ratios and cellular phenotypic outputs within a unified network framework, together with the integration of multi-model data stratified by tissue and disease stage, so as to move from “pathway-level associations” toward definable metabolic nodes and translatable therapeutic windows.

## 6 Therapeutic interventions targeting amino acid metabolic axes

The treatment of autoimmune diseases still relies mainly on pharmacological agents. At present, multiple drugs have been developed for the treatment of autoimmune diseases, some of which are capable of regulating macrophage metabolism and thereby affecting macrophage polarization. Hydroxychloroquine can inhibit iNOS levels in macrophages (109), downregulate M1 markers, and upregulate certain M2 markers, thereby promoting the transition of macrophages from the M1 to the M2 phenotype (110, 111). Glucocorticoids can suppress glycolysis by blocking the transcriptional and post-translational effects of hypoxia-inducible factor 1α (HIF-1α) in macrophages, while promoting glutamine metabolism to enhance TCA cycle flux and succinate metabolism, thereby preventing intracellular succinate accumulation and facilitating the conversion of macrophages from the M1 to the M2 phenotype (112, 113). Azathioprine and the calcineurin inhibitor cyclosporin A can suppress iNOS in macrophages, thereby inhibiting M1 polarization (114). Another calcineurin inhibitor, tacrolimus, can promote macrophage polarization toward an M2-like phenotype (115).

From the perspective of clinical translation and application, strategies aimed at “targeting amino acid metabolism to remodel myeloid/macrophage function” have advanced most rapidly in the field of cancer immunotherapy and most clearly illustrate a feasible path for metabolic intervention. Taking arginine metabolism as an example, myeloid cells in the tumor microenvironment, including tumor-associated macrophages and myeloid-derived suppressor cells, often suppress effector immunity through ARG1/ARG2-mediated arginine depletion. The oral small-molecule inhibitor INCB001158 (CB-1158), targeting this axis, has entered first-inhuman phase I studies and has been explored in combination with PD-1 inhibitors, suggesting that “relieving substrate depletion-driven immunosuppression” is clinically feasible from a safety and pharmacodynamic perspective, although its therapeutic efficacy remains to be fully established (116). Preclinical studies further support that arginase inhibition can alleviate myeloid cell-mediated immunosuppression and improve antitumor immune responses, thereby providing mechanistic and translational support for clinical combination therapy (117). With regard to glutamine metabolism, inhibition of glutamine utilization has likewise been incorporated into the clinical development strategy of “metabolic targeting plus immunotherapy.” Animal studies have shown that JHU083, a prodrug of the glutamine antagonist 6-diazo-5-oxo-L-norleucine (DON), not only suppresses tumor growth, but also reshapes the transcriptional profile and antigen-presentation/inflammatory cytokine characteristics of tumor-associated macrophages, shifting them from an immunosuppressive tendency toward a functional state more favorable for antitumor immunity (118). Consistent with this, the GLS inhibitor telaglenastat (CB-839) has entered phase I/II combination studies with immune checkpoint inhibitors such as nivolumab, reflecting a typical translational closed loop of “amino acid metabolism-targeted drug–combination immunotherapy–clinical safety and efficacy signal evaluation” (119). Overall, experience from the oncology field suggests that amino acid metabolism intervention is more likely to be implemented through well-defined clinical development strategies, including monotherapy or combination therapy, dose escalation, safety monitoring, and immune pharmacodynamic endpoints, and may provide a useful framework for trial design and stratification in immune-mediated diseases.

In autoimmune-related diseases, more direct human evidence has emerged for amino acid metabolite-receptor axes, represented by the Trp derivative-AhR pathway. In ulcerative colitis, for example, Indigo naturalis has completed multicenter randomized controlled trials, suggesting that oral treatment can induce clinical responses, while also emphasizing the need for rigorous safety assessment (120); animal studies have shown that this agent can ameliorate mucosal inflammation and local immune cell functional states through the AhR axis (121). In autoimmune diseases, amino acid metabolism-targeted interventions place greater emphasis on “reducing pathological inflammation and promoting tissue repair,” and therefore the available evidence is better organized from the perspective of feasible strategies and safety boundaries. First, regulation of the partitioning of arginine between iNOS and Arg1 has been shown in the arthritis setting to alter the inflammatory output of tissue macrophages and affect disease phenotypes (122), suggesting that the “arginine pathway node-synovial macrophage function-arthritis phenotype” axis is amenable to intervention. Oral L-arginine intervention can suppress inflammation in mouse models of RA (88), but systemic administration may simultaneously enhance the functions of certain pro-inflammatory cell populations. Because systemic alteration of substrate availability may affect multiple immune cell types, future efforts will require lesion-targeted delivery and patient stratification. Second, metabolic anaplerosis and nutritional interventions have relatively strong operability in mucosal immunity and chronic inflammatory settings. For example, α-KG supplementation can promote the transition of macrophages from M1 to M2/reparative phenotypes and improve disease activity in colitis models (123), providing support for the clinical research hypothesis of “nutrition-metabolic anaplerosis-immune repair.” Lowering circulating BCAA levels can inhibit M1 polarization, and modulation of their metabolic enzymes has become a therapeutic strategy in patients with obesity and insulin resistance (27). AhR agonists can markedly alleviate colitis in mice, providing strong support for clinical translation (42, 124). In mouse inflammatory models, AABA can attenuate disease phenotypes together with downregulation of pro-inflammatory macrophage phenotypes, but such evidence currently derives mainly from inflammatory or injury models, and its reproducibility and clinical applicability in classical autoimmune diseases still require further validation (67). Finally, because autoimmune diseases often require long-term medication and are subject to clear limitations related to systemic toxicity, new delivery approaches, such as local sustained-release systems and nanocarriers, are more suitable as “amplifiers and safety valves” for amino acid metabolism interventions, enabling the shaping of more favorable macrophage phenotypes within target tissues while reducing the risk of systemic exposure. Such strategies, when combined with existing immunosuppressive drugs or biologics, may also become a practical approach to improving long-term efficacy and reducing toxic side effects (125). Among these approaches, mesenchymal stem cell-derived exosomes (MSC-EXO) have attracted particular attention. Because of their low immunogenicity and good biocompatibility, exosomes have been widely studied for the treatment of clinical diseases. In recent years, increasing attention has been paid to the physiological and pathological functions of exosomes and their complex components. Recent studies have shown that exosomal crosstalk mechanisms may influence pathways related to immune responses, barrier function, and the gut microbiota (126, 127). Other studies have shown that exosomes can regulate a variety of amino acid-related receptors and inflammation-related cytokines (128–130), and whether a connection exists between these two aspects is also an issue that urgently requires further investigation.

To systematically summarize the current potential therapeutic strategies targeting amino acid metabolism to regulate macrophage polarization, **Table 1** outlines representative agents, their targets, their effects on macrophage polarization, and their stages of development. Because clinical studies directly targeting the amino acid metabolism-macrophage axis in autoimmune diseases remain limited, some translational evidence at the current stage is still derived mainly from pharmacological studies of the same targets in tumors or broader inflammatory diseases. Therefore, **Table 1** also includes preclinical and cross-disease translational evidence with mechanistic relevance. Amino acid metabolism has demonstrated a comprehensive range of therapeutic strategies in autoimmune disease treatment, spanning pharmacological intervention and nutritional modulation, as well as new target exploration and novel material-based delivery systems. The controllable regulation of macrophage immune function through multilevel metabolic reprogramming is likely to become an important direction for the future treatment of autoimmune diseases.

**Table 1:** Potential therapeutic interventions targeting amino acid metabolic axes and their research progress in inflammatory or autoimmune diseases.

Stage of development	Specific drug/strategy	Drug target	Experimental findings	Reference
Phase I clinical trial (solid tumor)	INCB001158 (CB-1158)	Arg1/2	Suppresses myeloid/macrophage-mediated immunosuppression and favors pro-inflammatory myeloid functional remodeling	(116)
Phase I/II clinical trial (combined with nivolumab; melanoma/renal cell carcinoma/NSCLC)	Telaglenastat (CB-839)	GLS1	Remodels the immune microenvironment; when combined with immunotherapy, it is used to improve myeloid/macrophage-associated immunosuppression	(119)
Clinical RCT (UC)	Indigo naturalis	AhR agonism	Favors reparative/anti-inflammatory effects	(120)
Preclinical (animal)	JHU083 (DON prodrug)	Broad-spectrum glutamine antagonism	Promotes a pro-inflammatory phenotype	(118)
Preclinical (animal)	α-KG supplementation	TCA cycle anaplerotic intermediate	Improves colitis phenotype	(123)
Preclinical (animal + human relevance)	Fra-1/Arg1 axis	Arg1 partitioning regulation	Alters macrophage inflammatory output and affects arthritis phenotype	(122)
Preclinical (*in vitro* + animal)	Serine restriction	Ser availability → mTOR → IL-1β	Suppresses the output of inflammatory macrophages, including IL-1β	(131)
Preclinical (*in vitro* + animal)	AABA	Amino acid-related metabolic reprogramming	Suppresses M1 inflammatory programs and limits inflammation-related disease phenotypes	(67)

This table summarizes recent drugs or intervention strategies targeting amino acid metabolism-related pathways, including regulatory approaches directed at metabolic axes such as arginine (Arg), tryptophan (Trp), glutamine (Gln), and serine (Ser), as well as their potential roles in the regulation of macrophage polarization and inflammatory responses. Different strategies influence pro-inflammatory (M1) or anti-inflammatory/reparative (M2) macrophage phenotypes by modulating key metabolic enzymes, nutrient-sensing signals, or metabolite levels, and have shown certain therapeutic potential in a variety of inflammatory or autoimmune disease models.

## 7 Discussion

In recent years, studies of immunometabolism have made it clear that amino acids are not merely substrates for protein synthesis, but are also embedded as multilevel metabolic nodes within the macrophage metabolic network, jointly shaping macrophage functional states through multiple layers, including substrate partitioning, TCA cycle anaplerosis, nutrient-sensing signals such as mechanistic target of rapamycin (mTOR) and GCN2, one-carbon/methyl donor cycling, and redox homeostasis (13). In the context of autoimmune-related chronic inflammation, multi-omics and metabolomic studies have consistently suggested that dysregulated amino acid metabolism shows cross-disease commonality and is often accompanied by skewed macrophage polarization, persistent inflammation, and tissue injury (1). Therefore, analyzing amino acid metabolism within the framework of the overall metabolic network may help explain the differential immunological effects of the same metabolic pathway across different tissue microenvironments, stimulus types, and disease stages, and may also provide a more causally meaningful analytical path for identifying key intervenable nodes (2).

At the level of the metabolic network, amino acids such as arginine, tryptophan, and glutamine can be regarded as key interfaces connecting multiple metabolic modules. Through substrate competition between iNOS and Arg1, arginine couples inflammatory NO production with polyamine/reparative metabolic branches, thereby regulating the balance between pro-inflammatory cytotoxicity and tissue repair within the same substrate framework (16). After entering the Kyn pathway through IDO1, tryptophan alters local nutrient availability through substrate depletion on the one hand, and shapes immune tolerance and inflammatory tension through Kyn and its receptor signaling on the other, causing this pathway to exhibit predominantly immunoregulatory functions in multiple autoimmune diseases, although its specific effects remain clearly dependent on the tissue microenvironment and the spectrum of microbiota-derived metabolites (2). Glutamine, by contrast, is metabolized into α-KG to achieve TCA cycle anaplerosis, and further couples mitochondrial metabolic status with epigenetic demethylation reactions, allowing changes in metabolic flux to be translated into stable alterations in macrophage polarization programs (26).

Taking TCA cycle reprogramming as an example, pro-inflammatory activation is often accompanied by TCA cycle disruption and the accumulation of signaling intermediates, among which succinate has been shown to promote IL-1β production and amplify inflammatory responses through HIF-1α (15). At the same time, itaconate alters succinate oxidation by inhibiting succinate dehydrogenase and exhibits anti-inflammatory effects in multiple models, thereby forming a dynamic regulatory circuit of “metabolite-enzyme activity-inflammatory signaling” (132). These findings suggest that, when evaluating the effects of amino acid-related metabolic axes, such as Gln→α-KG, on macrophage polarization, the key is not to simply classify them as “pro-inflammatory” or “anti-inflammatory,” but rather to focus on their integrated effects on anaplerotic flux, the spectrum of signaling metabolites, and the supply of epigenetic substrates, thereby reshaping inflammatory thresholds and reparative capacity across different timescales (26).

Nutrient-sensing pathways provide important clues for explaining the discrepant findings among different studies. For example, branched-chain amino acids (BCAAs) promote inflammation-related metabolic programs through transporters and mechanistic target of mTORC1 (mTORC1) signaling, whereas under conditions of amino acid deprivation or energetic stress, starvation sensing mediated by GCN2 can restrict inflammasome activation and IL-1β production, causing “supply” and “restriction” to generate opposite immune outputs at different thresholds (54). In human monocytes/macrophages, the amino acid transporter SLC7A5 has been shown to participate in immunometabolic reprogramming and to influence the intensity of inflammatory responses, suggesting that differences in transporter expression may be one of the key factors underlying tissue specificity and interindividual heterogeneity (18). Therefore, the functional divergence observed in BCAA-related studies is more likely to arise from “differences in nutrient-sensing thresholds” jointly shaped by multiple factors, including dose, time window, tissue substrate availability, and macrophage lineage origin, rather than from a simple directional change in a single metabolic pathway (11).

In addition, one-carbon metabolism and redox homeostasis also constitute important regulatory layers of macrophage polarization. Amino acid metabolism can alter the chromatin accessibility of inflammation-related genes by influencing SAM availability and methylation potential, thereby forming a regulatory loop of “metabolism-epigenetics-phenotypic stability” in chronic inflammatory environments (11). At the same time, the GSH/H_2_S system related to sulfur-containing amino acids can regulate ROS buffering capacity and mitochondrial function, and may exert tissue-protective effects in some inflammatory settings while producing immunosuppressive effects under specific conditions, thereby exhibiting marked environmental dependence (133). Therefore, the interpretation of related findings should return to verifiable key variables, including dose and time window, tissue niche, cell lineage origin, as well as substrate competition and transporter expression levels (2).

From the perspective of the evidence hierarchy, current studies on amino acid metabolism-mediated regulation of macrophage polarization are still derived mainly from *in vitro* polarization models and animal experiments. *In vitro* systems are useful for identifying key metabolic nodes and their threshold effects, but differences in amino acid concentrations in culture media, stimulation conditions, and criteria for polarization assessment may all affect the extrapolatability of the conclusions. Animal models are better able to reflect the integrated effects of tissue substrate availability and cell-cell interactions on metabolic flux, whereas human studies are inevitably influenced by multiple factors, including diet, gut microbiota, drug exposure, and comorbidities (134). In recent years, the establishment of cross-tissue single-cell atlases has systematically revealed the lineage and functional heterogeneity of human monocytes/macrophages in health and disease, providing an important reference framework for analyzing metabolism-phenotype coupling at the human level (135). At the same time, the molecular connections between inflammatory signaling and metabolic reprogramming have gradually been identified. For example, phosphorylation of signal transducer and activator of transcription 3 (STAT3) at Ser727 has been shown to be an important node through which toll-like receptor 4 (TLR4) mediates metabolic reprogramming and promotes IL-1β production, thereby providing a new mechanistic entry point for integrating “receptor signaling-metabolic flux-inflammatory output” (136).

At the translational level, interventions targeting amino acid metabolism have the advantage of simultaneously affecting multiple inflammatory pathways, but their clinical application still faces challenges such as systemic side effects and insufficient tissue specificity. In recent years, strategies aimed at restoring the balance between inflammation and repair by regulating macrophage metabolic reprogramming have been proposed in disease models such as lupus nephritis, providing new evidence for the feasibility of metabolic intervention in autoimmune diseases (59). At the same time, genome-scale metabolic network modeling based on clinical tissue data can identify key metabolic modules and predict priorities for potential interventions at the tissue level, thereby providing a systematic tool for translating correlation-based studies into mechanism-driven therapeutic strategies (137). In the future, the integration of metabolomic readouts, single-cell and spatial transcriptomic localization, and metabolic modeling analysis is expected to establish a research framework of “metabolic stratification-target selection-efficacy prediction,” thereby reducing the risk of failed extrapolation across different diseases or populations (138).

Overall, research on amino acid metabolism is moving from single-pathway mechanisms toward multi-node network regulation integrated with human validation chains. Future studies need to move beyond the linear explanatory framework based on a single metabolite or a single enzyme and systematically clarify, from the perspectives of temporal resolution and tissue niche resolution, the cooperative relationships between different amino acid metabolic axes and the TCA cycle, OXPHOS, and other immunometabolic modules (11). In addition, the dependence of macrophages on metabolic pathways is not completely consistent across different disease contexts, and the same metabolic alteration may correspond to different functional outputs in different tissues or at different stages of disease. Therefore, the effects of amino acid metabolism need to be interpreted in conjunction with specific pathological settings, which is also one of the major obstacles currently facing the clinical translation of immunometabolism research (9, 23). It should be noted that the immunoregulatory effects of amino acid metabolism are not limited to macrophages themselves. Amino acid availability, transporter expression, and metabolic sensing mechanisms such as mTOR and GCN2 can also indirectly shape the immune microenvironment in which macrophages reside by affecting the functional states of other immune cells, such as T cells. Therefore, it is necessary to investigate amino acid metabolism and macrophages at a broader scale (139). At the methodological level, isotope tracing technologies can directly resolve actual metabolic flux changes, single-cell and spatial multi-omics can help identify cell-cell interactions and substrate competition in the tissue microenvironment, and genome-scale metabolic modeling can be used for priority ranking and assessment of targetability. The integration of these three approaches at the level of the same sample will become an important direction for advancing this field from mechanistic research toward clinical translation (140). At the clinical strategy level, the establishment of quantifiable metabolic readouts, such as the Kyn/Trp ratio, the expression of key metabolic enzymes or transporters, and the proportions of signaling metabolites, combined with stratified interventions based on disease stage and tissue involvement, may help further promote tissue repair and restoration of immune homeostasis while suppressing inflammatory responses, thereby enabling treatment strategies for autoimmune diseases with greater long-term benefit (139).
